# Non-Ovarian Menstruation; with Cases and Some Remarks upon the Ovulation Theory

**Published:** 1870-10

**Authors:** A. Reeves Jackson

**Affiliations:** 1026 Wabash Ave.; Chicago, Ill.


Article II.—*Non-Ovarian Menstruation; with Cases, and Some Remarks upon the Ovulation Theory.* By A. Reeves Jackson, M.D., Chicago, Ill.

It has long been known that in many febrile conditions, and under a variety of circumstances quite unconnected with menstruation, there may be a bloody discharge from the uterus, and Mr. Spencer Wells* has proposed to distinguish these discharges from menstruation by the terms "Uterine Epistaxis," or "Metrostaxis."

* *Medical Times and Gazette*, Vol. II, 1866.

Ovariotomists especially have observed, too, that within a few days, sometimes within a few hours, after the operation of ovariotomy, there appears a sanguineous discharge from the vulva, which has also commonly been referred to the same class of cases. Even in the instances where these latter have returned with a certain degree of regularity, physiologists have never regarded them as functional, and I believe that to Dr. H. R. Storer is due the credit of having first called attention to the physiological importance of phenomena of this character.*

In the following case a discharge of the kind alluded to continued after the removal of *both ovaries*, and yet by its periodical return and persistent character it seems to present a fair claim to be regarded as menstrual. I would not feel justified in attaching this kind of importance to the case, however clear and direct the evidence it furnishes, did it stand alone. But it is corroborated by a number of others, the details of some of which have been kindly given me by Drs. Baker Brown, Atlee, and Storer, and which are hereto appended.

CASE I. Sarah T——, unmarried, aged forty-four years, commenced menstruating at the age of seventeen, and the process was always painlessly and regularly performed. August 31, 1865, I removed both ovaries, together with a portion of the Fallopian tubes.†

After her recovery, which was rapid and complete, she was frequently under my observation, being a resident of the same town. Some time during the year 1866 I learned that she had been menstruating regularly since the operation — a circumstance which surprised me very greatly, although my patient, an uneducated woman, was not aware that the fact was at all singular. On making close inquiry I was informed that a menstrual period ceased on the 30th of August, 1865. The operation was done in the afternoon of the following day. October 1, 1865, after an interval of thirty-one days, the discharge again appeared, and continued four days, attended by the usual symptoms of lassitude,

* *American Journal of the Medical Sciences*, January, 1868, p. 81.

† A detailed account of this case was published in the *American Journal of the Med. Sciences*, Vol. LII, p. 111, where, however, a typographical error makes the patient's age forty-seven nervousness, backache, etc. There now occurred an interval of eighty-three days, the discharge reappearing December 22, 1865. Its next appearance was January 20, 1866—four weeks after—and from this last date it had continued to return with entire regularity, every twenty-eight or twenty-nine days, accompanied by all the ordinary symptoms of menstruation, and lasting each time from three to five days.

I now watched the case with much interest, and ascertained that the regularity above noted continued down to October, 1867, —a period of twenty-two months. It then ceased until February, 1868, when it appeared for the last time, the lady being then forty-seven years of age.

I may add that during all this time the patient's general health was excellent, and that I satisfied myself on several occasions that there was no disease of the uterus or vagina. Her voice has become harsher and more masculine, but otherwise there is no change discoverable.

I communicated the principal features of this case to Drs. W. L. Atlee, Spencer Wells, Charles Clay, Baker Brown, H. R. Storer and others, and was surprised to find that they had each met with similar ones, some of which I am permitted to refer to.

CASE II. (W. L. Atlee.) "Mrs. J. C., aged thirty-six years, mother of six children. Menstruation always returned five months after parturition. Menstruated regularly the last time, March 20, 1854.

"April 17, 1854, both ovaries were removed at eleven o'clock, A. M. Menstruation returned the same evening.

"On the anniversary of the operation for several years, Mrs. C. regularly wrote to me, keeping me advised of her condition, always assuring me that the menses continued to return regularly.

"Dec. 5, 1867, I saw the patient. She informed me that up to May 1, 1864, she continued to menstruate as regularly as she had ever done. It then ceased. In May, 1865, she had a return, which was the last of it. Dec. 3, 1866, she was 48 years old.

"August 1, 1868, I called to see her while passing through B——, and noticed that she had a *shaved beard*."

CASE III. (W. L. Atlee.) "Miss K. V., aged nineteen years, has menstruated regularly, accompanied with some dysmenorrhœa. Had a regular menstruation just four weeks ago. April 25, 1855, removed both ovaries.

"October 25, 1855, I saw the patient. She had just issued her wedding cards. She has had the regular monthly molimen as before the operation, and also a *monthly discharge*, but not stained with blood.

"She married, made a visit to Europe, and after her return I learned from her mother that the monthly discharge continued, and that she had the proper enjoyments of matrimonial life."

Dr. H. R. Storer, of Boston, has placed upon record three cases of double ovariotomy, and *in all of them* there was a subsequent reappearance of the menses.

CASE IV. (H. R. Storer.*) "Sarah A. Colcord, aged 47, unmarried. The menses have always been normal, though somewhat scanty. Sept. 23, 1865, both ovaries and the whole of the uterus, except a small portion of the cervix, were removed.

"From the date of the operation until October 11, eighteen days subsequently, and twenty-six days after the last appearance of the catamenia, there was no discharge whatever from the vagina. Then there occurred a sanguineous effusion, attended by feelings of lassitude, backache, etc., etc., lasting thirty hours, and being an evident attempt at the re-establishment of menstruation; a very curious circumstance, and of great physiological interest, when it is recollected that the uterus and both ovaries had been removed."

CASE V. (H. R. Storer.†) Mrs. Dunham, aged 43 years, the mother of six children. Menstruation had been regular down to within two months, and was now suppressed, as was supposed, by pregnancy. The symptoms became urgent, and Nov. 20, 1867, both ovaries were removed. "In the present instance the menses had been absent for two months, and yet reappeared subsequently to the operation, although the ovaries had both been removed, and the major part of the Fallopian tubes also."

* *American Journal of the Med. Sciences*, January, 1866, p. 110.

† *Canada Medical Journal.*

CASE VI. (H. R. Storer.*) Mrs. Matthews. "The ovaries were entirely removed, and yet the patient has had, quite regularly, a sanguineous discharge, without evidence of uterine disease, and which hæmostatics, generally and locally applied, have failed to check or prevent."

Dr. Charles Clay, of Manchester, England, informs me (April 24, 1869,) that in about 220 cases of ovariotomy, he has performed double ovariotomy four times, and that in three of them he had noticed subsequent sanguineous discharge, which, however, he had never considered as menstrual, but attributed it to congestion of the lining membrane of the uterus. He adds, "I must confess the evidence in your case is remarkably strong, and if correct would entirely upset all the usual theories of menstruation."

Dr. I. Baker Brown, in a letter dated April 19, 1869, says, "I think I have had about five cases similar to your own in 148 cases of ovariotomy, but in some of them I could not *completely* remove *all* the disease."

CASE VII. (Baker Brown.) "A little more than two years ago I operated on a young lady *extremely* reduced by ovarian (multilocular) dropsy, and removed both ovaries, and yet she has ever since menstruated, but not regularly—sometimes missing a few weeks."

CASE VIII. (Baker Brown.) "The case on which I lay most stress was one on which I operated about ten years ago. I used the clamp externally, and the stump of the pedicle adhered to the parietes. At every subsequent epoch the skin broke, and she menstruated through the stump, and some through the vagina."

Le Fort also reports a case similar to that of Miss T., in the *Gazette Hebdomadaire.*

Koeberle records a case of this kind also, in which not only the ovaries but a large portion of the uterus were removed, *without interfering with the menstrual flux.*†

Notwithstanding the fact that the ovulation theory of menstrua-

* *American Journal of the Med. Sciences,* January, 1868.

† Liegois on the "Function of Reproduction," being part 1st of his "Traitè de Physiologie appliquè a la Medecine et a la Chirurgérie." Paris, 1869.

tion in the human female has been generally accepted by physiologists and accoucheurs as that which seemed most fully and satisfactorily to account for all the phenomena of that process, it is yet not unlikely that they may be obliged to modify their views in some important particulars.

Cazeaux, after considering the matter fully, says, "Having read all that has been written on the subject, the mind rests satisfied in its ability to refer this singular phenomenon to one unchangeable and easily verified fact, namely, the successive evolution of the Graafian vesicles.

"That the cause of the menstrual discharge is the evolution of a Graafian vesicle, would be an indisputable proposition, provided we were able to show—1st, That the examination of women who died during or shortly after the menstrual period had uniformly revealed the above-named changes; 2nd, That the absence of ovaries involved, of necessity, the absence of menstruation; 3rd, and lastly, That there is a complete analogy between the anatomical and physiological phenomena of the heat of animals and those which accompany menstruation (the menstrual flow) in the human female."

The observations and experiments of Coste, Negrier, Pouchet, Raciborski and others, which have been thought to prove the first of these points, cannot be considered as at all conclusive; and the statement made by Cazeaux that "no one has succeeded in instancing the case of a single woman who died at the menstrual period, whose ovary did not present a vesicle in a greater or less degree of development, or else one which had been already ruptured," may be admitted as entirely true, and yet cannot be taken as evidence of any necessary connection between ovulation and menstruation. For the researches of the elder Ritchie, confirmed more recently by those of the son,* demonstrate most clearly that Graafian vesicles, in successive stages of development, are to be found in the ovaries from early childhood to advanced age without having any connection with, or influence in determining, that process.

* Contributions to assist the Study of Physiology and Pathology. By Charles G. Ritchie, M.D., London, 1865.

The conclusions of Dr. Ritchie are so important in relation to this subject that I give them in full:

"1. The Fallopian tubes of the infant, and of the child before puberty, are perfect in their structure, although their patency is more or less obstructed at birth by the presence in them, and in the uterus, of a tenaceous, glue-colored mucus.

"2. The ovaries of new-born infants and children are occupied, sometimes numerously, by Graafian vesicles or ovisacs, which are highly vascular as early as the sixth year, and vary in size from the bulk of a coriander seed to that of a small raisin, in the fourteenth year, at which time, also, they are filled with their usual transparent granular fluid.

"3. The Graafian vesicles contained in the ovaries prior to menstruation are found, as they also are in every other period of life, in continual progression towards the circumference of the glands, which they penetrate, discharging themselves by circular-shaped capillary-sized pores or openings in the peritoneal coat; the presence of the catamenia being thus no indispensable prerequisite to their rupture.

"4. The establishment of menstruation does not necessarily give rise to any immediate modification in the manner in which the ovisacs are discharged, or in the subsequent change which these bodies undergo; but in some cases the conditions which obtain in the period before puberty, are extended for a time into that of menstruation.

"5. The ovisacs of the human female do not require the establishment or presence of menstruation for their development or rupture. Vesicles of adult size may exist and be discharged about the age of puberty, as also at other periods of life, independently of menstruation; and this state may be present in its normal form for at least eight consecutive periods without a vesicle being ruptured, unless after the manner and with the phenomena which occur in childhood.

"6. In early infancy, extreme old age, and long-continued organic disease, the ova are minute, transparent and structureless cells; and in advanced childhood, during the critical age, and during pregnancy and lactation, they are more or less organized, larger, and in the latter state are often so well matured, that about one-third of the renewed pregnancies of married women take place while they nurse.

"7. In children and others in the circumstances now mentioned, the exercise of organic power which occasions the secretion and extrusion of ova, is attended also by that of an opaque mucus in the tubes and uterus; but on the attainment by the female of maturity, and onward to the period of critical life (when the animal powers become again diminished, and the calibre of the arteries reduced), the ovarian orgasm, like many other vital actions, undergoes periodical augmentations of power, during which, unless when prevented by the disturbing influence of other functional processes or by disease, it extends to the nervous and vascular tissues of the uterus, and gives rise to the formation within this viscus of decidual vessels, which exude lymph and red globules, the latter being evacuated, mixed with watery fluid, mucous matter, and some of the salts of the blood, in the form of menstruation.

"8. That the periodical congestion or increase in the size of the ovaries before and at the usual menstrual terms is not caused by the pressure or bursting of ripe vesicles, is plain from the consideration that the ovaries are often crowded with such, some of which also occasionally give way, and empty themselves by capillary-sized openings in the surface of the glands in women who have never menstruated; and in others who have ceased to menstruate, one and two large and fully-developed vesicles are frequently seen.

"9. The elimination of ova, and the process of menstruation, are correlative effects of the vital powers of the ovaries (just as the secretion of mucus and gastric juice, and the chymification of the blood, are those of the stomach); and to suppose the rupture of the Graafian vesicles to be the cause of the menses, is to mistake a frequent association, and, to some extent, effect, for a uniform cause.

"10. The principal use of menstruation is, in regard to the ovary, to provide an accessory by which the maturation of its vesicles, and the absorption of their peritoneal and tenacious proper coats, and their extension generally, may be promoted throughout the child-bearing period of life; and, in reference to the uterus, to furnish a nidus within its walls by which the ovum may be entangled, retained and nourished."

From all this it would seem clear that ovulation cannot be regarded either as the cause or effect of menstruation; and, furthermore, that however generally the two processes may accompany each other, this accompaniment is by no means constant or essential. And facts are not wanting to substantiate the correctness of these positions.

For example, Rondelet mentions the case of a woman who was delivered twelve times, and Joubert another, who bore eighteen children, neither of whom had ever menstruated.* And many instances have been related of girls who have become pregnant prior to the appearance of menstruation, and of others who have become so long after menstruation had ceased—all tending to show that ovulation may exist quite independently of the menstrual flow.

On the other hand, a case in which a discharge apparently identical with the menstrual fluid took place every fourteen days during three successive pregnancies, is given by Graily Hewitt in the *London Lancet* for July 24, 1858, p. 91.

Cazeaux admits that the menstrual flow may sometimes appear without necessarily implying the actual rupture of a matured Graafian vesicle, but he explains such cases on the ground that the matured vesicle, having established the menstrual movement, may remain stationary for some time, and finally abort without rupture.

It is needless to say that this explanation is entirely unsupported by facts. The extensive observations of M. Cazeaux have furnished him with undoubted evidence that the discharge of an ovule is not always necessary to the menstrual discharge, but, wedded to the theory that considers the vesicular evolution the only cause of the flow, he is forced to the assumption that in such cases the process has been half completed, half left undone.

But while many of the advocates of the ovulation theory are willing to admit that the ovule may maturate and even be discharged without the presence of the menstrual flow, they yet insist that when menstruation does occur we may feel entirely satisfied as to the existence of the vesicular development; that is to say, they deny that menstruation can occur except as an epiphenomenon of ovulation. And, hence, they argue, menstruation,

* Gardien, *Traitè d' Accouchements*, Vol I, p. 220.

although not a cause of fecundity, is yet a sign of it. But it is plain that if menstruation may occur in a woman who has no ovaries, it can no longer be regarded as a sure indication of fertility.

In order that I may not be misunderstood I wish to say that the cases of alleged menstruation occurring during pregnancy have never found favor with me. I have always thought that close investigation would reveal that the characteristic marks of natural menstruation had been for the most part absent; that the discharge had not returned with the usual regularity as to time; that it had not continued uninterruptedly the usual number of days; that it had differed in quantity and quality from the true menstrual fluid; and, lastly, that the discharge had not been accompanied by the feelings that are commonly experienced by females during normal menstruation.

It is quite true that when we consider the open state of the Fallopian tubes and the cervix uteri up to the second month of pregnancy, and the condition of the decidua vera and decidua reflexa, it is not difficult to understand that they may readily permit the menstrual discharge, in this early stage of pregnancy, being derived from its ordinary source, the lining membrane of the cavity of the uterus. This might be the source of the flow until the cavity of the uterus becomes completely occluded by the contact and coalescence of the decidua vera and reflexa which take place in the third month. The cervical plug of mucus is the only obstacle to its issuing from the uterus. But this is also present in the unimpregnated organ, and requires to be displaced or opened up for each ordinary menstrual period.

And now since we have seen that in some anomalous cases a small portion of the cervix uteri may be sufficient, as in the cases of Storer and Koeberle, to furnish a regular persistent discharge having all the characteristics of the normal menstrual flow, and attended by the usual accompaniments of the latter, we may not find so much difficulty as heretofore in comprehending how such discharge may continue throughout the entire period of utero-gestation.

The appeal to comparative physiology for the purpose of giving support to the ovulation theory is, I think, an unfortunate one. The œstrum of animals and menstruation in the human female are not completely analogous processes; there are indeed points of essential difference between them. In the former the period marks the *only* time when the male will be received, and the only time when his approaches would be effectual. Whereas there has always been a feeling of repugnance to the sexual act on the part of man, and a delicate shrinking on the part of woman, during the menstrual period. It is well known that among the Jews, and likewise among nearly all eastern nations, sexual intercourse is prohibited at such times by their religion, for the reason that it is "uncleanness." It is also an admitted fact that although impregnation in the human female is much more likely to occur just before, or within a few days after, a menstrual period, yet it is possible at any time. So that although we may admit the intimate and even necessary connection between œstruation and ovulation in animals, it by no means follows that the same relation exists between ovulation and menstruation in woman.

If then it can be shown, 1st, That there is *no* necessary connection between the successive evolution and extrusion of ova and the phenomena of menstruation; 2d, That the absence of ovaries does *not* necessarily imply absence of the menstrual flow; 3rd, That the œstruation of animals is *not* identical with the catamenial discharge; and, 4th, That ovulation and menstruation may each exist without the other, *can* we continue to maintain the correctness of the ovulation theory? I do not mean to be understood as contending that these points are established; for I have no wish to claim more importance for the cases and facts which have been given than fairly belongs to them. But if we give credence to them at all, it seems to me that we must admit that the presence of the ovaries and the process of ovulation are are not the *only* things necessary to menstruation or the menstrual molimen; that the lining membrane of the cavity of the uterus is not the *only* source of the menstrual discharge; and that the "ovulation" theory must be either materially modified, or else discarded as not sufficient to account for all the phenomena.

1026 Wabash Ave.

